# Supplementary material for: Amyloid-β42/40 ratio drives tau pathology in 3D human neural cell culture models of Alzheimer’s disease
Source: Nat Commun. 2020 Mar 13;11:1377. doi: 10.1038/s41467-020-15120-3 (PMC7070004; doi:10.1038/s41467-020-15120-3)
Supplement: Supplementary file 1 — Supplementary Information [file 41467_2020_15120_MOESM1_ESM.pdf]

Supplementary Information for

Amyloid-β 42/40 ratio drives tau pathology in 3D human neural cell culture models of Alzheimer's disease

By Kwak et al.

Supplementary Table 1

Heterogenous Parental cell lines	Homogeneous Single clonal lines	Expressed genes
ReN-G	ReN-G2#B2	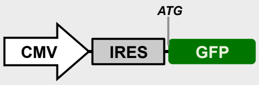
ReN-m	ReN-m3#C8	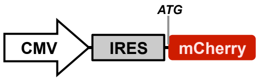
ReN-GA	ReN-GA2#A5	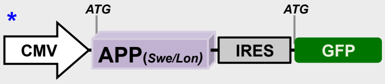
ReN-mGAP	ReN-mGAP10#D4<br>ReN-mGAP2#H10	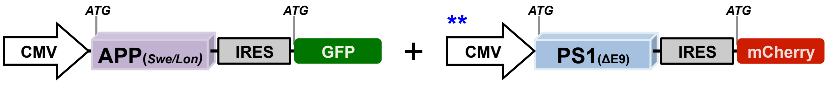
ReN-mAP4	ReN-mAP4#E6F4	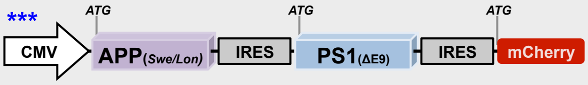
HReN-mGAP	HReN-#C2H2<br>HReN-#A4H1	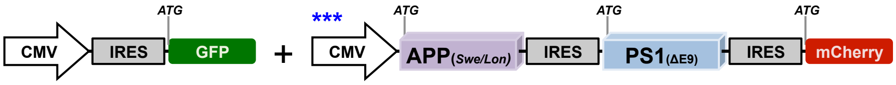

Supplementary Table 1. A list of lentiviral DNA constructs expressed in major clonal hNPCs. The expression levels of APPSL (*) determine total Aβ levels while PS1ΔE9 (**) levels affect Aβ42/40 ratio by altering PS1/γ-secretase complex in clonal AD hNPCs. In case of hNPCs with APPSL-PS1ΔE9 construct (***), the Aβ42/40 ratio can not be changed since transcription of both APPSL and PS1ΔE9 are controlled by a single CMV promoter.

Supplementary Figure 1

a

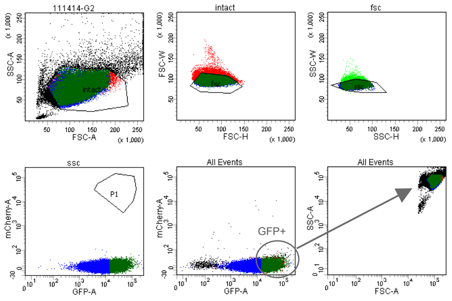

ReN-G

Population	#Events	%Parent	%Total
All Events	104,512	####	100.0
intact	99,039	94.8	94.8
fsc	95,277	96.2	91.2
ssc	93,244	97.9	89.2
mCherry+	0	0.0	0.0
GFP+	32,518	34.9	31.1
P1	0	0.0	0.0

b

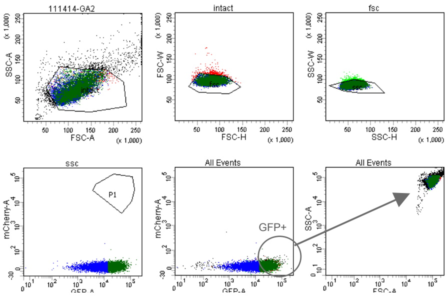

ReN-GA

Population	#Events	%Parent	%Total
All Events	12,690	####	100.0
intact	11,692	92.1	92.1
fsc	11,301	96.7	89.1
ssc	11,027	97.6	86.9
mCherry+	0	0.0	0.0
GFP+	4,672	42.4	36.8
P1	0	0.0	0.0

c

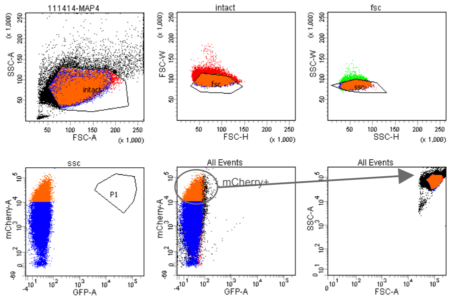

ReN-mAP

Population	#Events	%Parent	%Total
All Events	80,934	####	100.0
intact	73,901	91.3	91.3
fsc	69,152	93.6	85.4
ssc	66,909	96.8	82.7
mCherry+	13,288	19.9	16.4
GFP+	0	0.0	0.0
P1	0	0.0	0.0

d

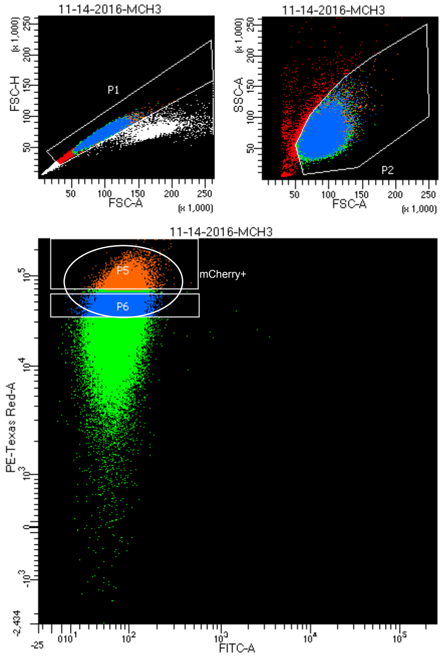

ReN-m

Population	#Events	%Parent	%Total
All Events	110,855	####	100.0
P1	102,367	92.3	92.3
P2	100,555	98.2	90.7
P3	0	0.0	0.0
P4	0	0.0	0.0
P5	8,819	8.8	8.0
P6	33,181	33.0	29.9

e

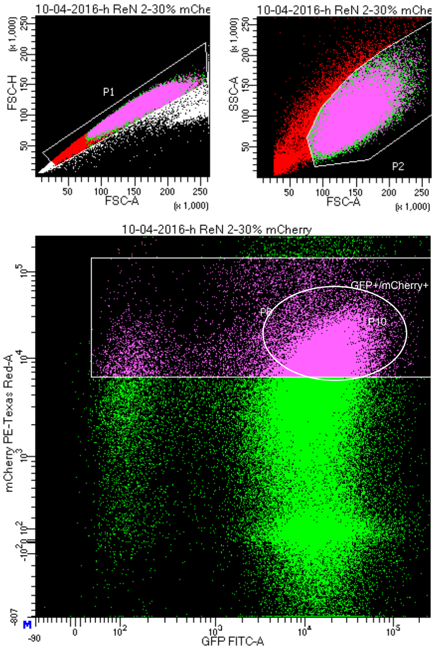

HReN-mGAP

Population	#Events	%Parent	%Total
All Events	130,000	####	100.0
P1	113,190	87.1	87.1
P2	105,343	93.1	81.0
P3	80	0.1	0.1
P4	849	0.8	0.7
P5	16	0.0	0.0
P6	29	0.0	0.0
P8	30,882	29.3	23.8
P10	22,026	20.9	16.9

Supplementary Figure 1: FACS-assisted single cell sorting by GFP and mCherry signals. a-e) Gating strategies applied in cell sorting processes for (**a**) ReN-G, (**b**) ReN-GA, (**c**) ReN-mAP, (**d**) ReN-m and (**e**) HReN-mGAP cell lines are presented.

Supplementary Figure 2

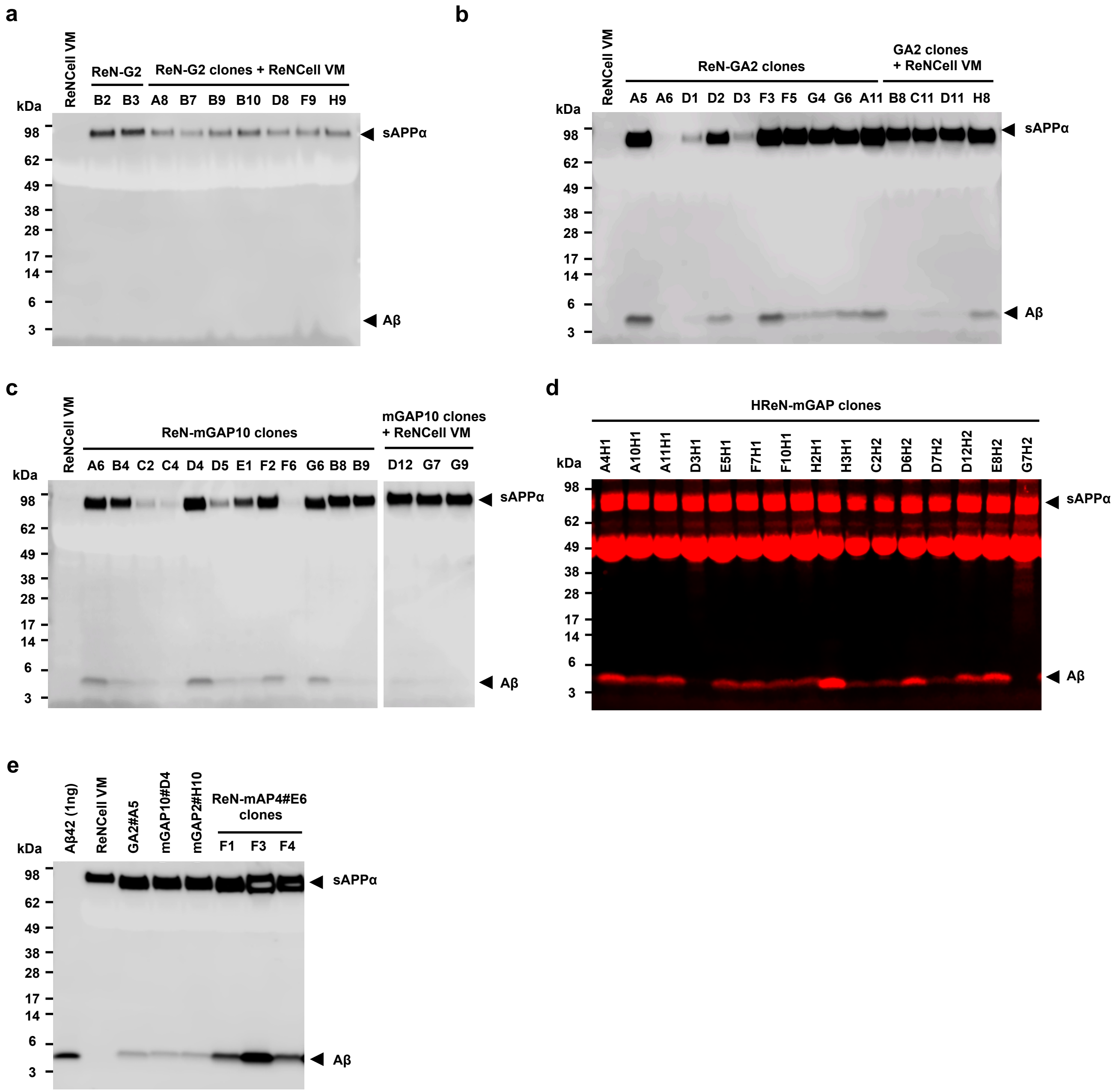

Supplementary Figure 2: Levels of Aβ in the conditioned media from clonal hNPCs. a-d) Single cells sorted by FACS were expanded in 96-well plates for two weeks. The conditioned media from each clonal hNPC was collected, then, levels of Aβ were analyzed by Western blot analysis. The code numbers for each clone from ReN-G2 (**a**), ReN-GA2 (**b**), ReN-mGAP10 (**c**)**,** and HReN-mGAP (**d**) are presented. (**e**) Single cell sorting protocol with the parental ReN-mAP4 cells was conducted. Levels of Aβ peptides in conditioned media from three clonal ReN-mAP4 cells, #E6F1, #E6F3, and #E6F4, respectively, were analyzed by Western blot analysis.

Supplementary Figure 3

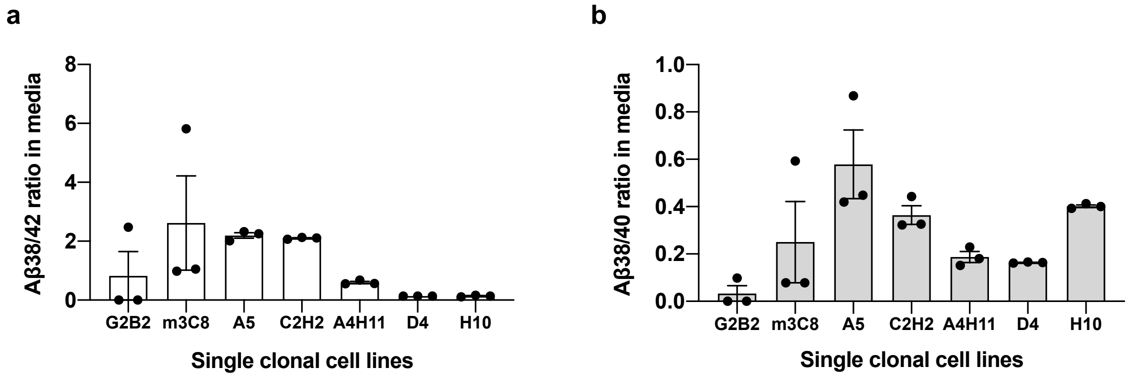

Supplementary Figure 3: Ratios of Aβ38/42 and Aβ38/40 in clonal hNPCs.

a,b) Ratios of Aβ38/42 (**a**) and Aβ38/40 (**b**) for the indicated clonal hNPCs were presented. All data were expressed as mean ± SEM of three independent repeats (black dots).

Supplementary Figure 4

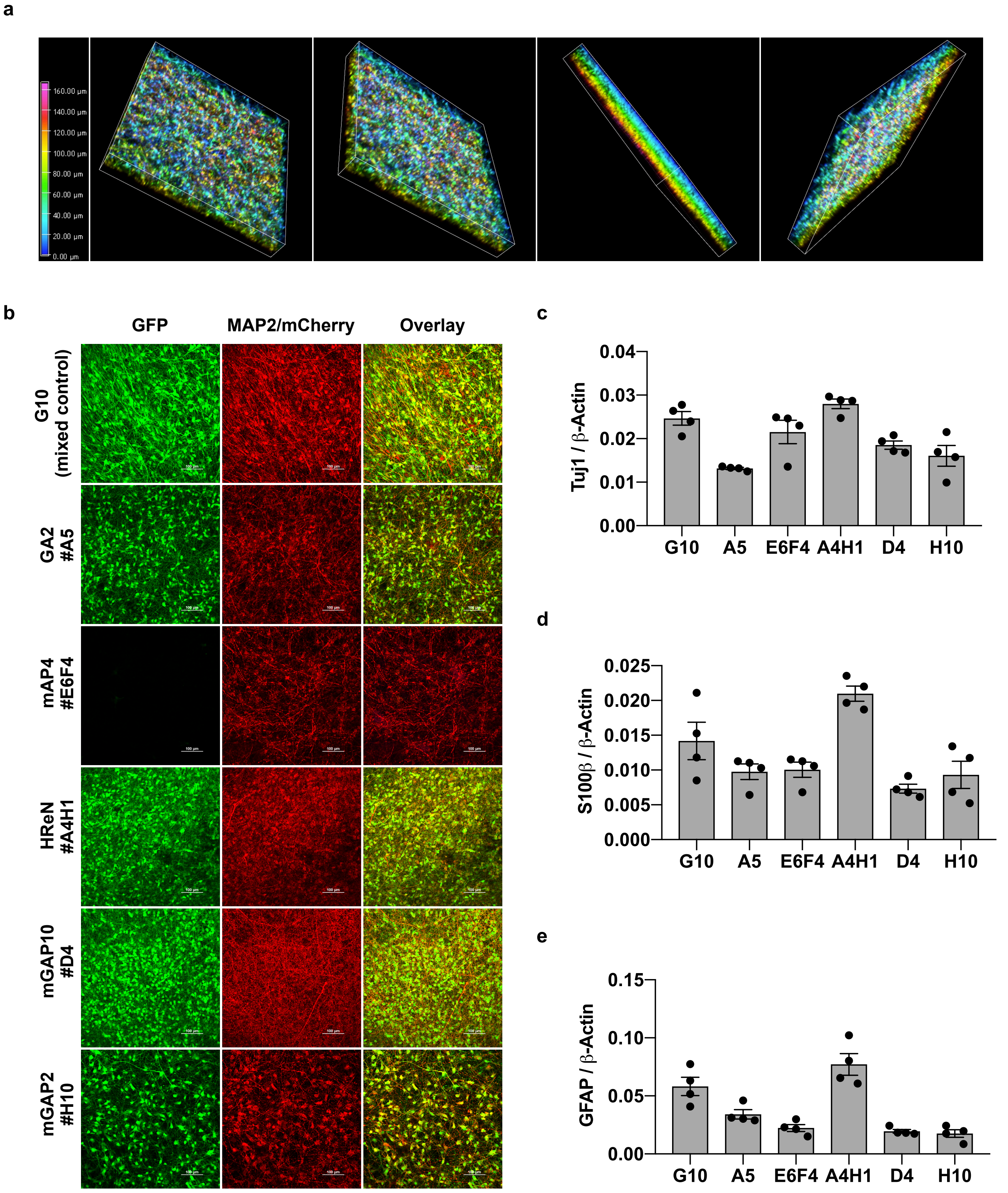

Supplementary Figure 4: Expressions of neural markers in 3D-differentiated clonal hNPCs for 5 weeks. a) Representative three-dimensional Z-stack images for the 3D culture are presented. **b)** Neural differentiation of clonal hNPCs in 3D culture. Representative cells plated in 3D gels were differentiated for 5 weeks and stained with anti-MAP2 antibody for differentiated neurons. The fluorescence signals and MAP2 positive cells were imaged by confocal microscopy. Scale bars represent 100 μm. **c-e)** Whole proteins were extracted from 3D-differentiated clonal FAD hNPCs, ReN-GA2#A5 (A5), HReN-mGAP#A4H1 (A4H1), ReN-mAP4#E6F4 (E6F4), ReN-mGAP10#D4 (D4), and ReN-mGAP2-#H10 (H10), as well as control cells, ReN-G10, in 96-well plates for 5 weeks. The neural markers such as Tuj1 for neurons (**c**) and S100β (**d**) and GFAP (**e**) for astrocytes were assessed by dot blot analysis. Levels of those markers were quantified by using Fiji software. Values were normalized by β-Actin levels. All Date were expressed by mean ± SEM of four independent repeats (black dots).

Supplementary Figure 5

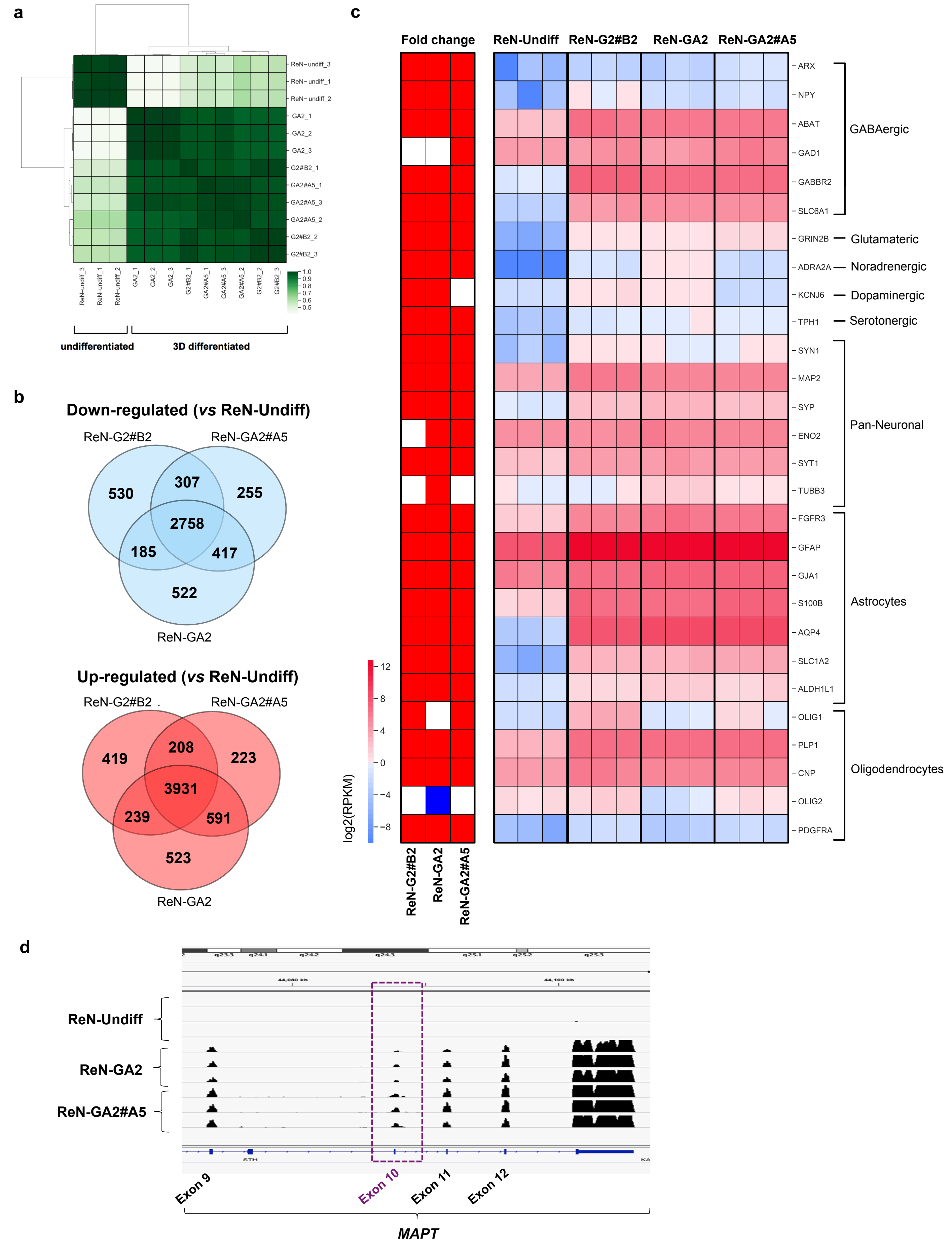

Supplementary Figure 5: Neural and glial markers in 3D-differentiated mixed and clonal hNPCs identified by RNA-sequencing. a) Pearson correlation of gene expression profiles (RPKM) from undifferentiated ReNcell VM cells and ReN-G2#B2, ReN-GA2, and ReN-GA2#A5 cells, differentiated for 7 weeks in 3D **b)** Venn diagram showing shared differentially expressed genes (FDR-adjusted p-value < 0.05 & |log2(fold change| > 1) for G2#B2, GA2#A5, and GA2 *vs* ReN-Undiff cells. Blue diagram = shared down-expressed genes (log2(fold change) < -1) as compared to ReN-Undiff. Red diagram = shared up-expressed genes (log2(fold change) > 1) as compared to ReN-Undiff. **c)** Heat map showing log2(RPKM) gene expression values for select neural and glial markers in undifferentiated and differentiated ReN cells. Fold change column (left) indicates which markers are significantly (FDR-adjusted p-value < 0.05) up-expressed (log2(fold change) > 1, red), down-expressed (log2(fold change) < -1, blue), or show no change in expression (white) for G2#B2, GA2, and GA2#A5 cells as compared to ReN-Undiff cells. **d)** A diagram presenting expression of *MAPT* exon 9, 10, 11 and 12 in mixed (ReN-GA2) and clonal AD hNPC model (ReN-GA#A5).

Supplementary Figure 6

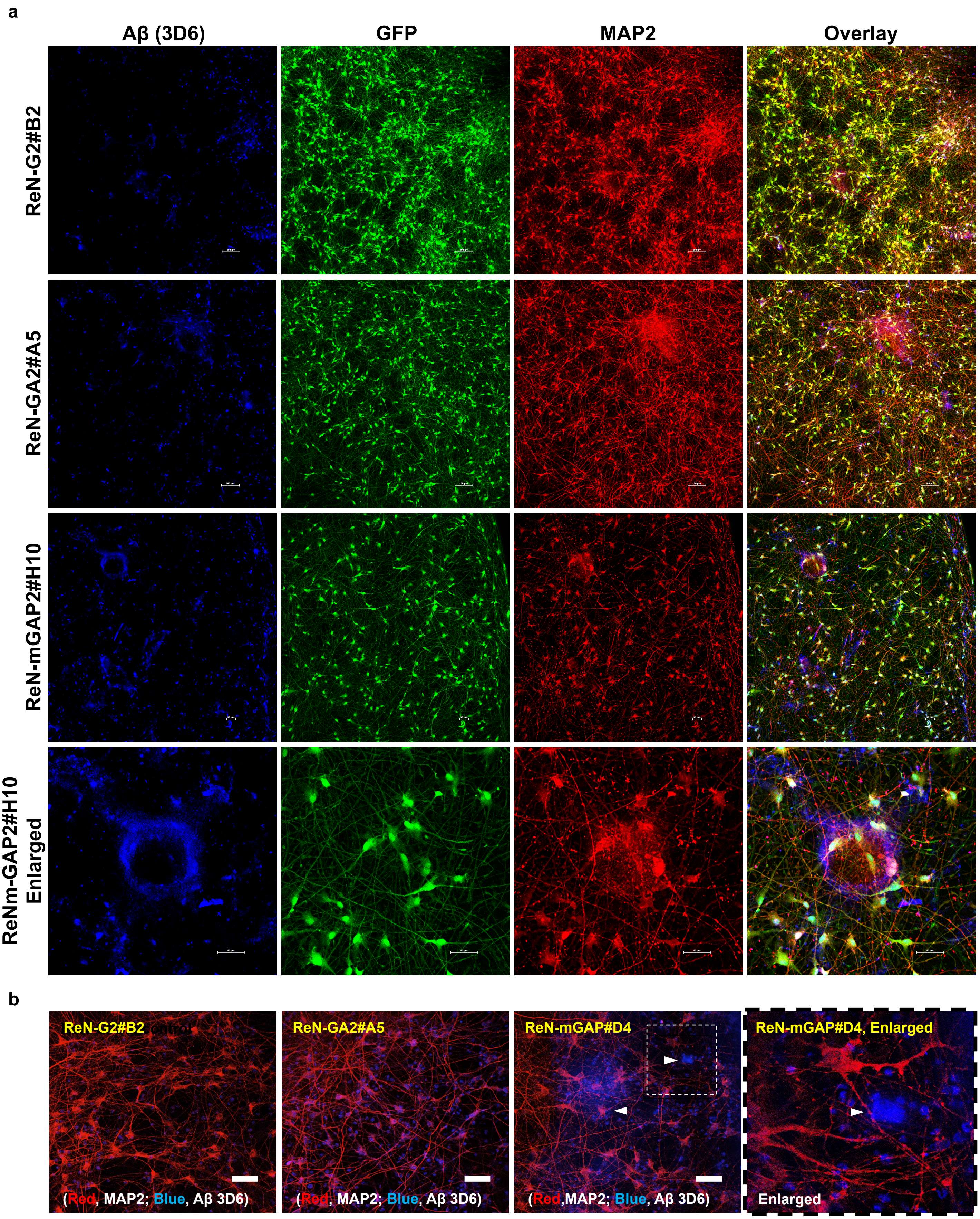

Supplementary Figure 6: Aβ42/40 ratio regulates Aβ accumulations in 3D cultures. a) Clonal control (ReN-G2#B2) and FAD hNPCs (ReN-GA2#A5 (low Aβ42/40 ratio) and ReN-mGAP2#H10 (high Aβ42/40 ratio)) (3×10^5) were 3D-differentiated in 96-well glass-bottom plates. After 6 weeks, cells were co-immunostained with antibodies against Aβ (3D6) and MAP2. Scale bars represent 100 µm. Enlarged images for ReN-mGAP2#H10 cells are presented. Scale bars for the ReN-mGAP10#D4 and its enlarged images represent 50 µm. **b)** Clonal control (ReN-G2#B2) and FAD hNPCs (ReN-GA2#A5 and ReN-mGAP#D4) (3×10^5) were 3D-differentiated in 96-well glass-bottom plates. After 6 weeks, cells were co-immunostained with antibodies against Aβ (3D6) and MAP2. Scale bars represent 500 µm. The enlarged image from ReN-mGAP10#D4 (square with white dot) is presented to show Aβ deposition-like structures.

Supplementary Figure 7

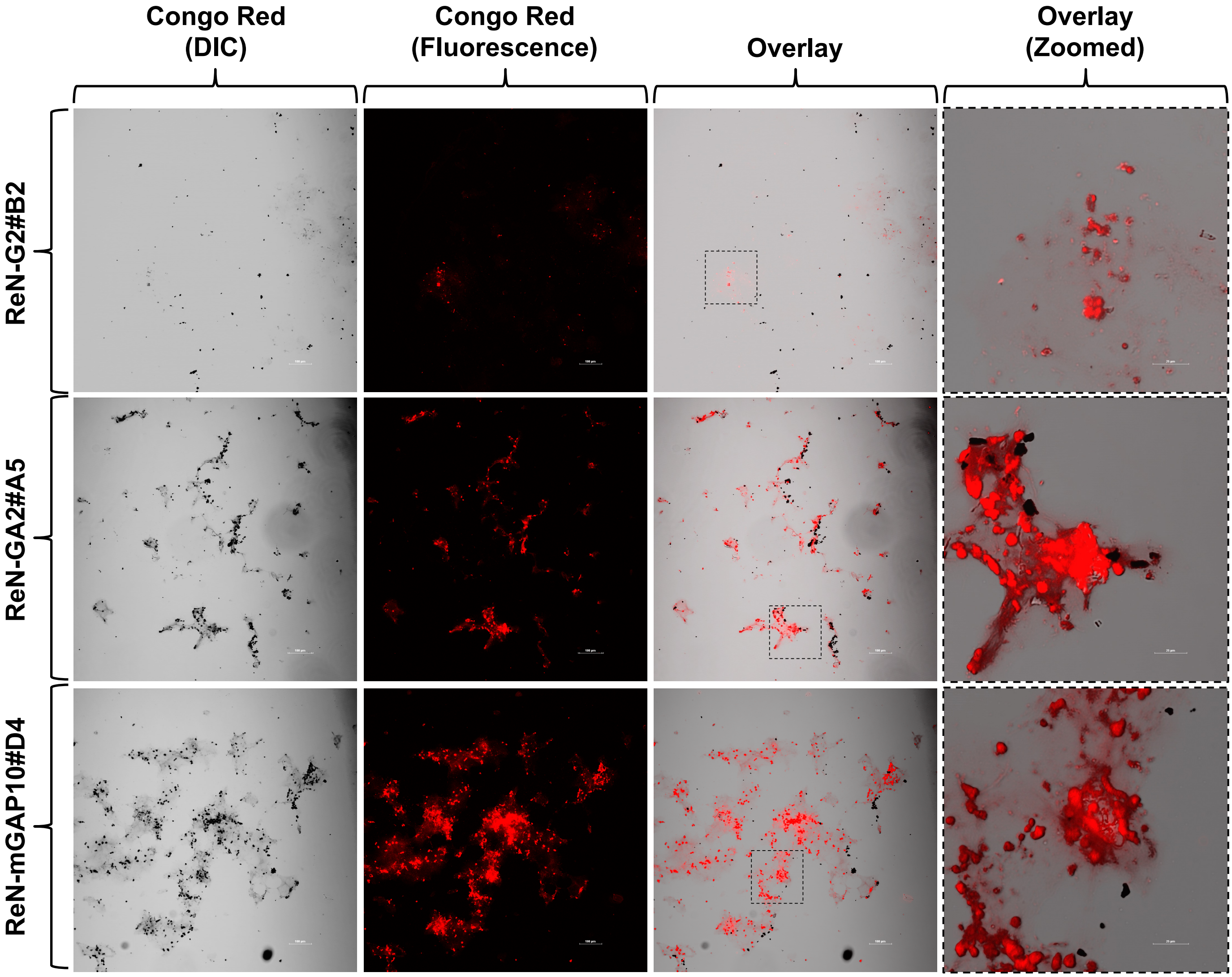

Supplementary Figure 7: Amyloid levels are robustly increased in clonal FAD hNPCs with high Aβ42/40 ratio. The 3D-thin layer cultures of ReN-G2#B2, ReN-GA2#A5 and ReN-mGAP10#D4 cells (8×10^6) were prepared in 6-well plates and differentiated for 9 weeks. Detergent-insoluble protein fractions were prepared by ultracentrifuge (300,000 × g, 30 min at 4˚C), which were stained with Congo red. The stained amyloids were examined by light microscopy. DIC indicates differential interference contrast. Scale bars represent 100 μm for regular images and 20 μm for zoomed insets.

Supplementary Figure 8

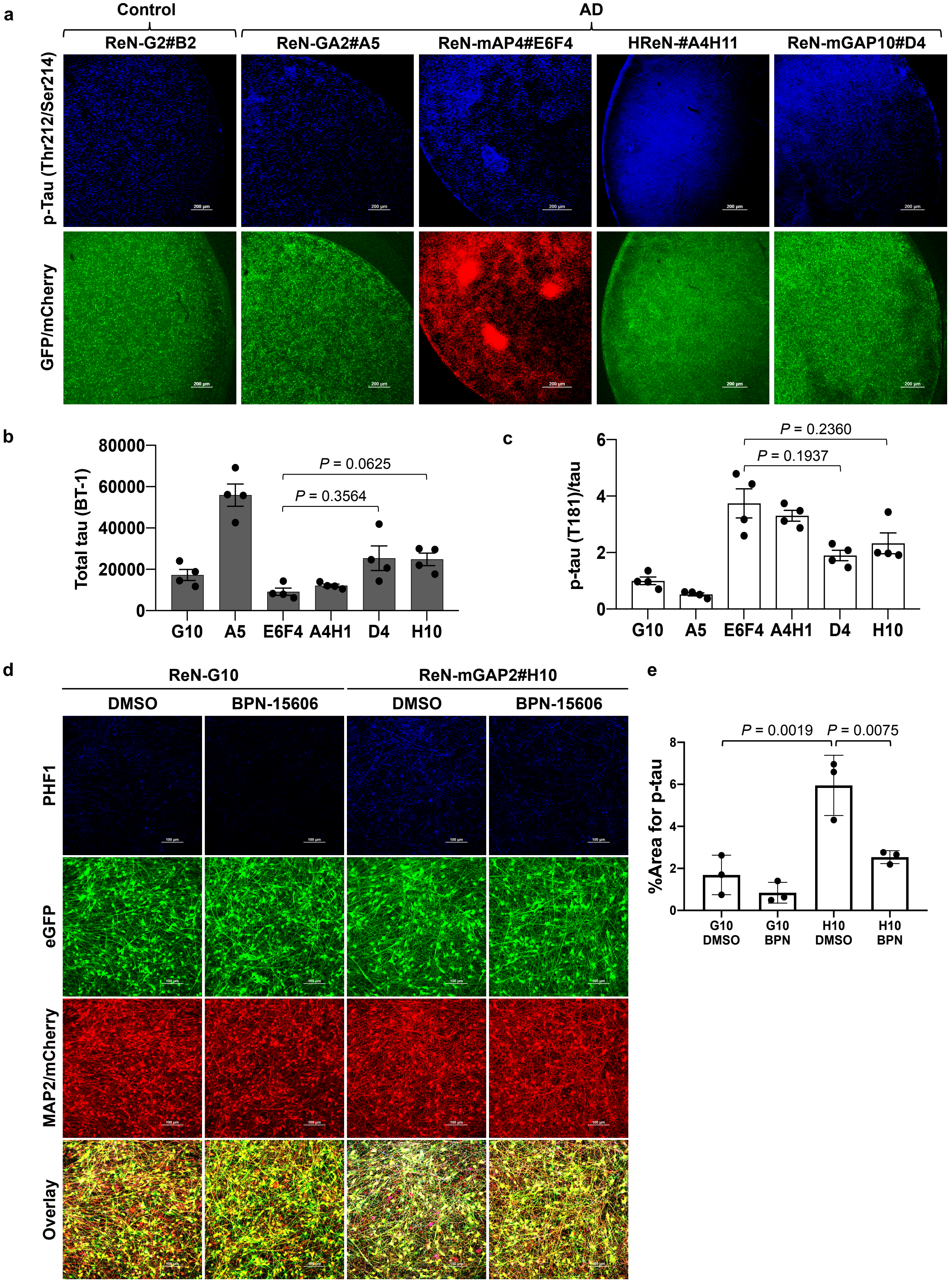

Supplementary Figure 8: Accelerated p-tau levels in 3D-differentiated FAD hNPCs with high Aβ42/40 ratio. a) Clonal control and FAD hNPCs (3×10^5) were 3D-differentiated in 96-well flat-bottom plates for 6 weeks. After fixing the cultures with 4% PFA, cells were immunostained with anti-p-tau antibody (AT100; Thr212/Ser214). The GFP and mCherry signals represent differentiated hNPCs in the 3D culture. Scale bars represent 200 μm. **b)** The protein samples from 6-week differentiated control (ReN-G10) and clonal FAD hNPCs (ReN-GA2#A5, ReN-mAP4#E6F4, HReN-mGAP#A4H1, and ReN-mGAP#D4) were prepared by 5M GuHCl lysis buffer. Using BT-1 antibody, levels of total tau were analyzed by ELISA assay. **c)** The ratio of p-tau-T181/tau was calculated using the values from Figure 4c and total tau. All data were expressed as mean ± SEM of four independent repeats (black dots). Statistical significances were determined by one-way ANOVA with Tukey's multiple comparisons test. **d)** Immunofluorescence analysis of the effects of chronic BPN-15606 treatments (70 nM) on p-tau accumulation in 6-week 3D-differentiated ReN-G10 or ReN-mGAP2#H10 cells. Levels of p-tau were analyzed by PHF1 immunofluorescence staining. MAP2 antibody was used as a neurite marker to monitor potential toxicity of BPN-15606. **e)** Accumulation of p-tau levels were quantified by using Fiji software. All values were combined to express as mean ± SEM of three independent repeats (black dots), Statistical significances were determined by one-way ANOVA with Tukey's multiple comparisons test.

Supplementary Figure 9

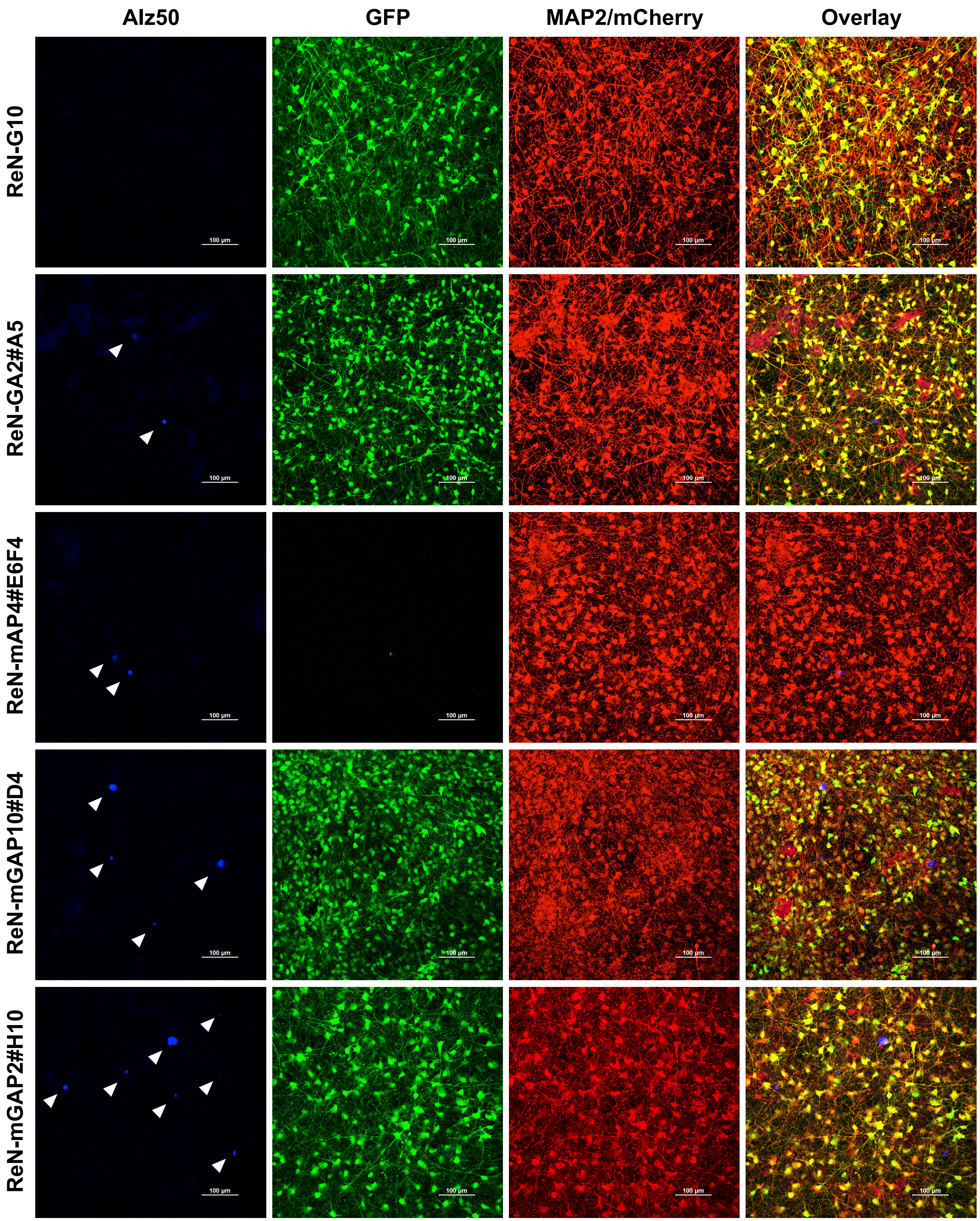

Supplementary Figure 9: Increased aggregated tau in 3D-differentiated FAD hNPCs with high Aβ42/40 ratio. Control (ReN-G10) and clonal FAD hNPCs (ReN-GA2#A5, ReN-mAP4#E6F4, ReN-mGAP10#D4, and ReN-mGAP2#H10) (3×10^5) were 3D-differentiated in 96-well flat-bottom plates for 6 weeks. After fixing the cultures with 4% PFA, cells were immunostained with Alz50 antibody against aggregated tau species and MAP2 antibody for differentiated neurons. The GFP signals represent differentiated hNPCs in the 3D culture. White arrow heads indicate the aggregated tau species detected by the Alz50 antibody.

Supplementary Figure 10

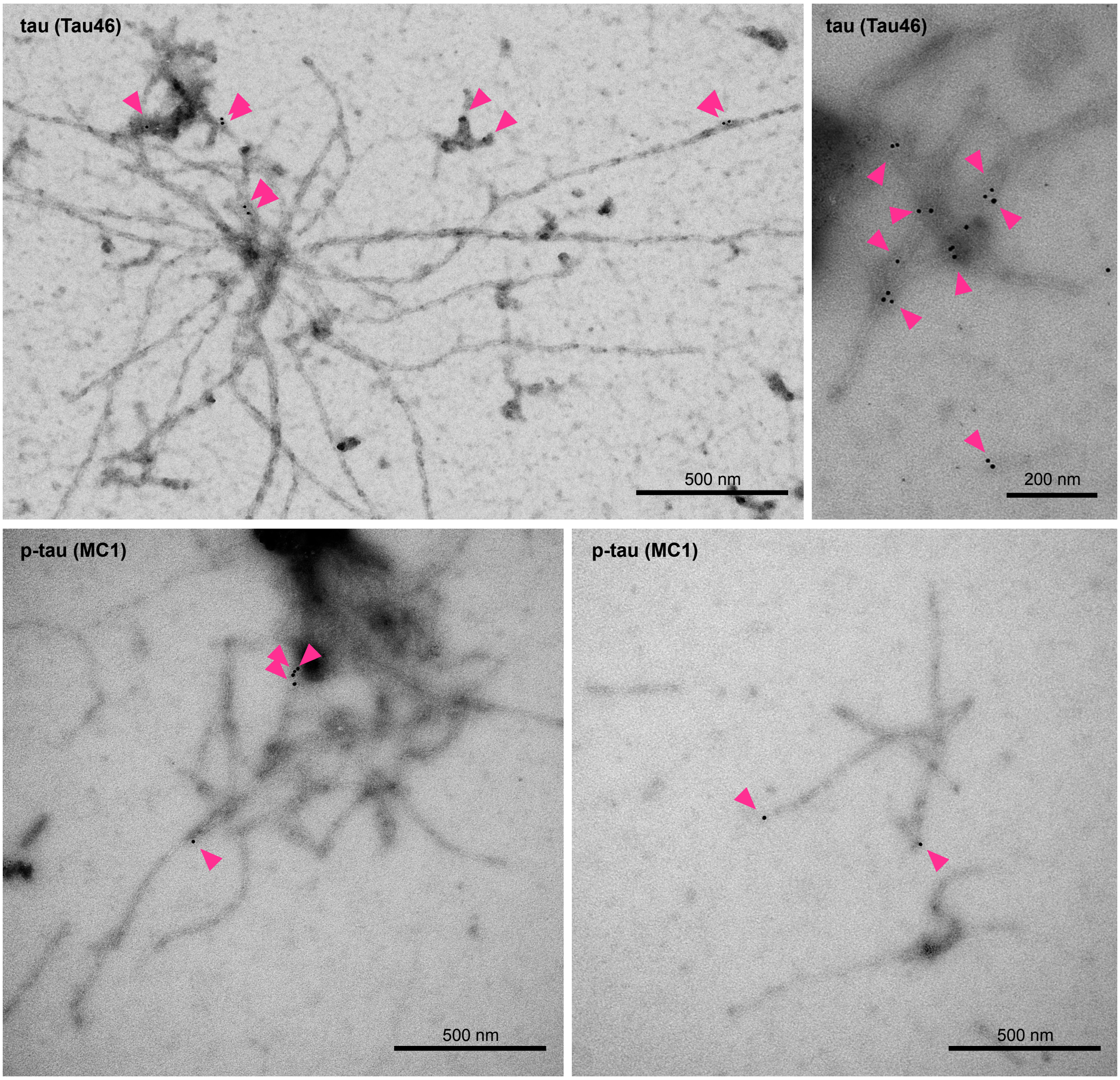

Supplementary Figure 10: Fibrillary structures tau proteins in detergent-insoluble fractions. ReN-mGAP10#D4 cells (8 × 10^6) were plated in 6-well plates and differentiated for 9 weeks. Detergent-insoluble fibrillary structures were prepared using ultracentrifuge (300,000 × g, 30 min at 4°C) followed by immunogold staining. The detergent-insoluble fibrillary tau proteins or p-tau were analyzed with Tau46 for tau and MC1 for p-tau, respectively. Red arrowheads indicate gold nanoparticles staining tau and p-tau. Scale bars are presented.

Supplementary Figure 11

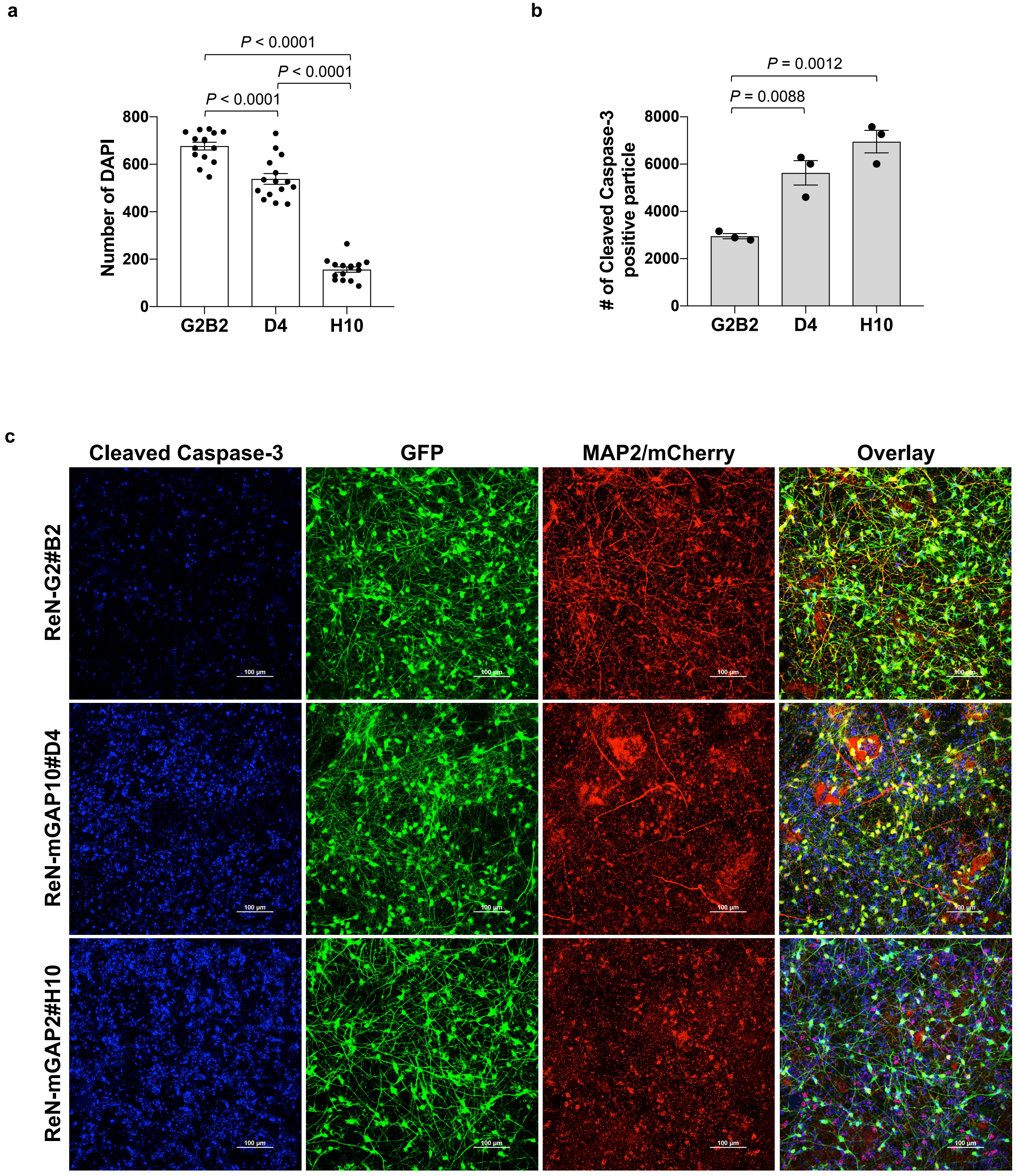

Supplementary Figure 11: Increased cell death correlates with high Aβ42/40 ratio in 3D-differentiated FAD hNPCs a) Counts of DAPI-positive cell in 3D cultures. ReNG2#B2, ReN-mGAP10#D4 and ReN-mGAP2#H10 cells (3×10^5) were plated in triplicate in 96-well flat-bottom plates and differentiated for 5 weeks. Triplicate samples from each of five separate plates were stained with the nuclear stain DAPI and counted using the Analyze Particles tool in Fiji software. Counts for each cell type are presented as mean ± SEM of fifteen independent repeats (black dots). Statistical significances were determined by one-way ANOVA with Tukey’s multiple comparisons test. **b, c)** ReN-G10 (non-AD control), ReN-mGAP10#D4, and ReN-mGAP2#H10 cells (3×10^5) were 3D-differentiated in 96-well flat-bottom plates for 6 weeks. Cells were fixed with 4% PFA followed by immunostaining with an antibody against cleaved/activated caspase-3. Fluorescence signals from apoptotic cells detected by the cleaved caspase-3 antibody were counted using the Analyze Particles tool in Fiji software. All values were combined to express as mean ± SEM of three independent repeats (black dots). Statistical significances were determined by one-way ANOVA with Tukey’s multiple comparisons test.

Supplementary Figure 12

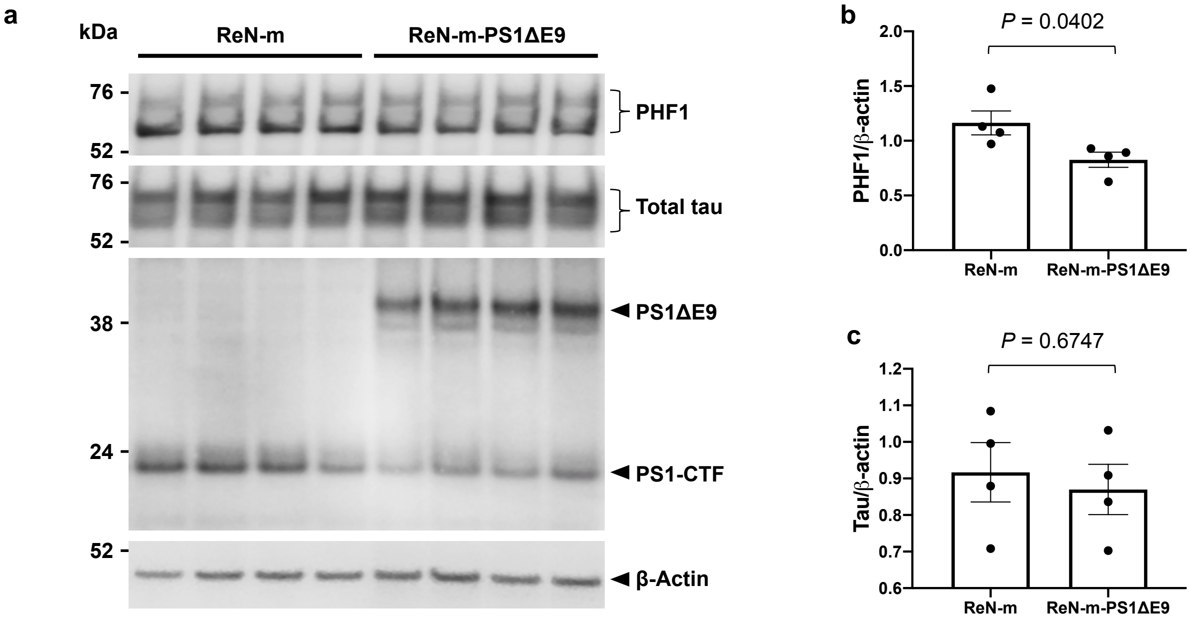

Supplementary Figure 12: Overexpressing PS1ΔE9 did not induce robust tau phosphorylation in 3D-differentiated hNPCs. a) Lentiviral DNA constructs harboring mCherry or PS1ΔE9-mCherry were transduced into the naïve hNPCs (ReNcell VM). Cells (1×10^6) were plated in 3D-thin layer cultures (24-well plate) and differentiated for 6 weeks. Western blot analysis was performed to compare the p-tau levels between ReN-m, expressing mCherry only, and ReN-m-PS1ΔE9, expressing PS1ΔE9-mCherry. **b, c)** Relative levels of p-tau (b) and total tau (c) were quantified by using Fiji software. All values were normalized with β-actin levels. The results were combined to express as mean ± SEM of four samples (black dots), Statistical significances were determined by unpaired two-tailed *t*-test.

Supplementary Figure 13

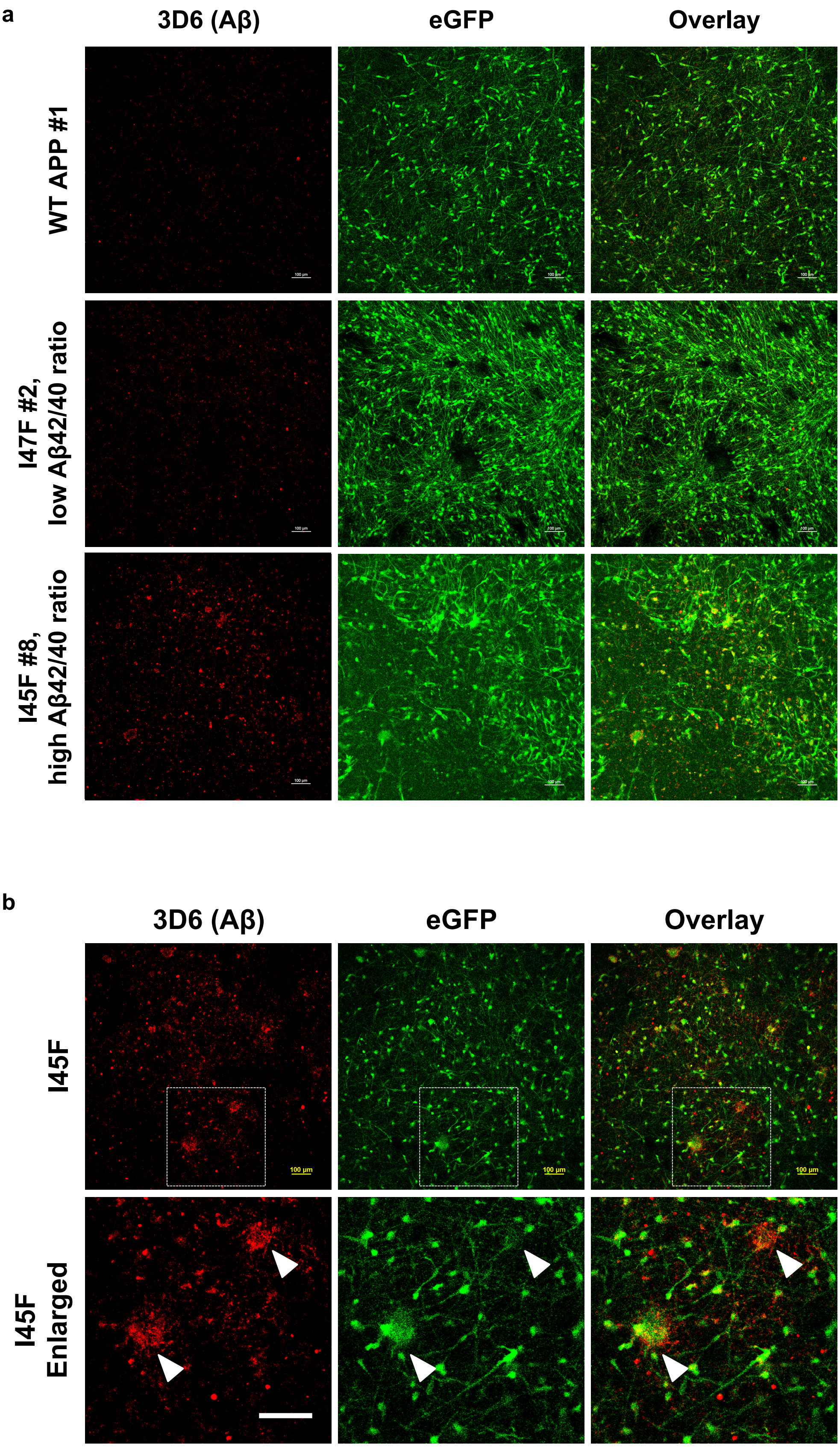

Supplementary Figure 13: Immunofluorescence analysis of Aβ accumulation in 3D-differentiated APP TMD hNPCs. a) APP TMD hNPCs (3×10^5) plated in flat bottom 96 well plates were 3D-differentiated for 7 weeks. After fixed with 4% paraformaldehyde, cells were immunostained with anti-Aβ antibody, 3D6. Confocal microscopic analysis reveals that APP TMD I45F cells boosted up the accumulation of Aβ in 3D gels as compared to APP WT or APP TMD I47F cells. Scale bars represent 100 μm. **b)** Different version of APP TMD I45F cells (3×10^6) exhibiting robust accumulation of Aβ were plated and 3D-differentiated for 7 weeks in flat bottom 96 well plates. The enlarged images (bottom) indicated aggregated Aβ peptides (white arrowheads). Scale bars represent 100 μm.

Supplementary Figure 14

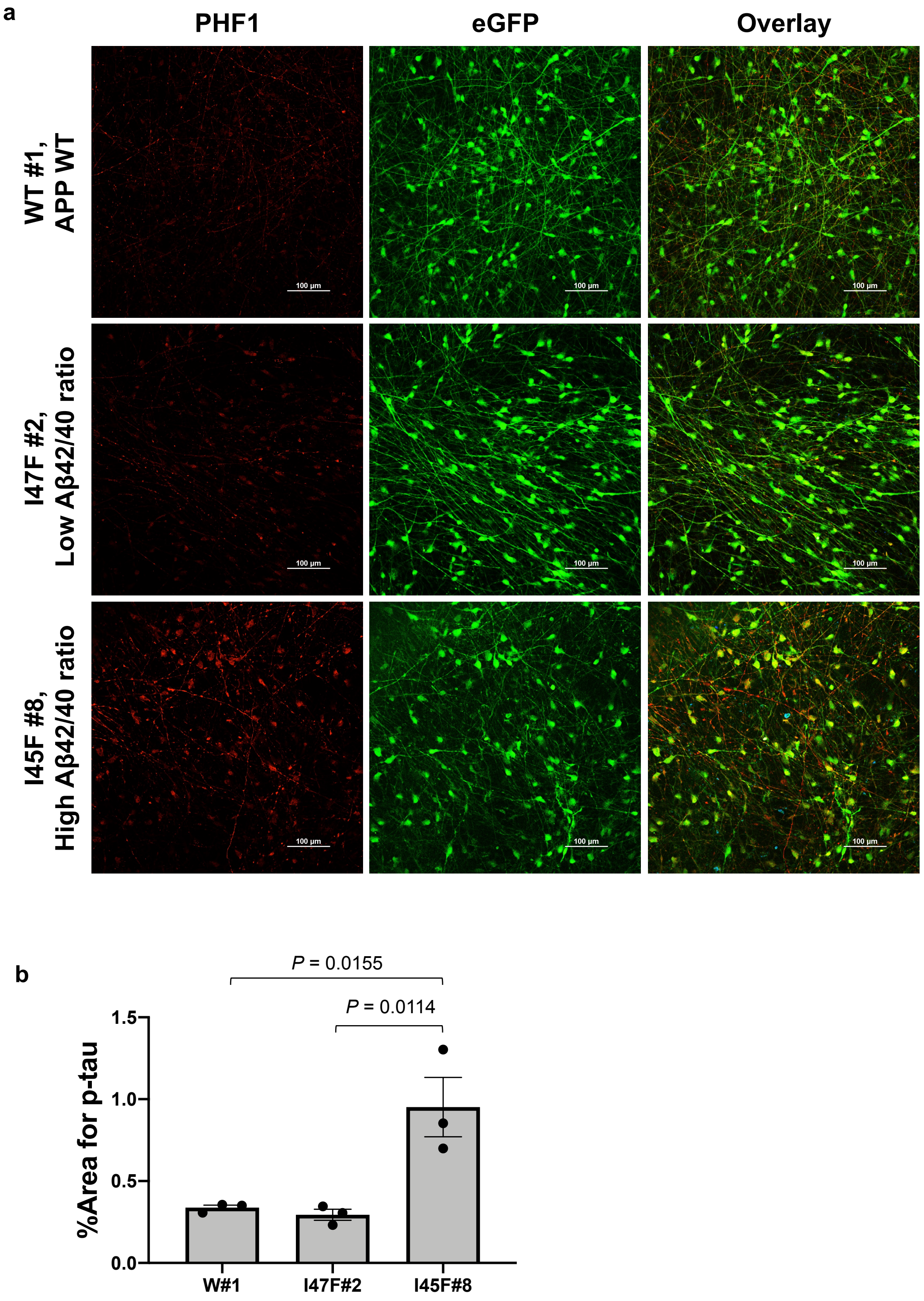

Supplementary Figure 14: Immunofluorescence analysis of p-tau accumulation in 3D-differentiated APP TMD hNPCs. a) APP TMD hNPCs (3×10^5) plated in flat bottom 96 well plates were 3D-differentiated for 7 weeks. After fixed with 4% paraformaldehyde, cells were immunostained with anti-p-tau antibody, PHF1. Increase of p-tau levels in APP TMD I45F cells were analyzed by confocal microscopic analysis. Scale bars represent 100 μm. **b)** Quantification of p-tau levels in 3D-differentiated APP TMD hNPCs. The percentage of p-tau signals in the total area was analyzed by using Fiji software. The results were combined to express as mean ± SEM of three independent repeats (black dots). Statistical significances were determined by one-way ANOVA with Tukey's multiple comparisons test.

Supplementary Figure 15

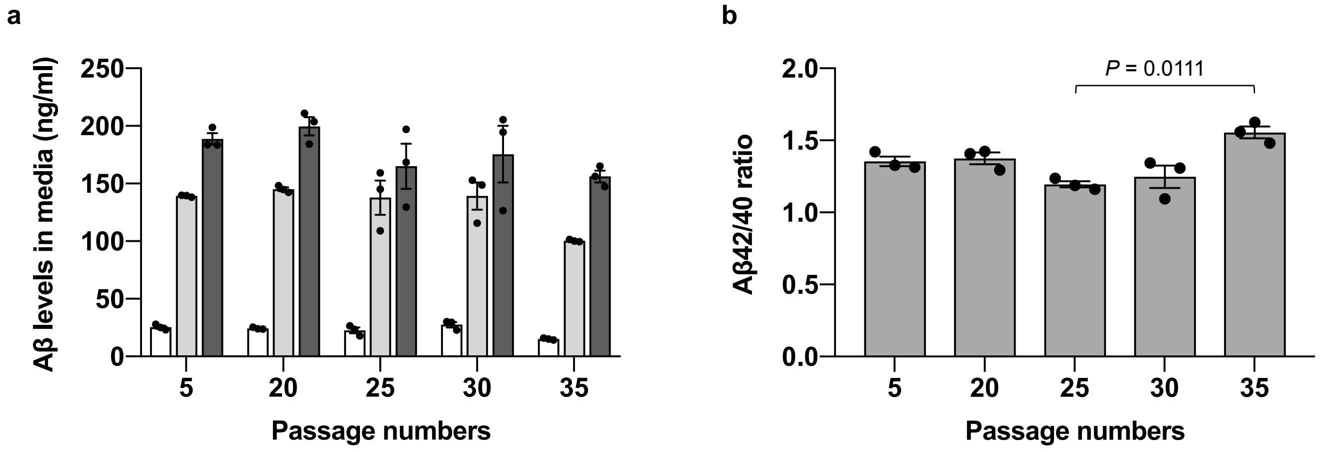

Supplementary Figure 15: Clonal FAD hNPCs can maintain high levels of Aβ and Aβ42/40 ratio even at high passages. a) Different passage numbers of ReN-mGAP10#D4 cells were prepared. Cells (3×10^6) were grown in Matrigel-coated 6-well plates for 24 h. After changing media with 1 ml of fresh expansion media, cells were incubated for an additional 24 h. Conditioned media was analyzed by MSD Aβ assay to measure concentrations of the secreted Aβ38, Aβ40 and Aβ42. **b)** Aβ42/40 ratios were determined based on the concentrations of Aβ species. All data were expressed as mean ± SEM of three independent repeats (black dots). Statistical significances were determined by one-way ANOVA with Tukey’s multiple comparisons test.
